# Interaction with the Paxillin LD1 Motif Relieves MEKK2 Auto-inhibition

**DOI:** 10.5334/1750-2187-10-4

**Published:** 2015-10-16

**Authors:** Michael P. Kahle, Bruce D. Cuevas

**Affiliations:** Department of Molecular Pharmacology and Therapeutics, Stritch School of Medicine, Loyola University Chicago, Maywood, IL, USA

**Keywords:** Kinase, phosphorylation, auto-inhibitory domain, scaffold, auto-phosphorylation

## Abstract

The cell signaling molecule MEK kinase 2 (MEKK2) is a key upstream regulator of MAPK activity that regulates numerous cellular functions, but the mechanisms that control MEKK2 activity are not well understood. Recently, we reported that MEKK2 both binds and promotes ubiquitylation of the scaffold protein paxillin, and thereby modulates the composition of adhesion complexes. In this study, we have extended our examination of this interaction and report that recombinant paxillin is sufficient to induce MEKK2 auto-phosphorylation. Furthermore, we utilize siRNA-mediated paxillin expression knockdown to reveal that MEKK2 activity is reduced in paxillin-deficient cells. Finally, we show that the paxillin leucine-rich motif 1 (LD1) is sufficient to bind to the MEKK2 amino terminal region and activate MEKK2. Taken together, our results show for the first time that paxillin association promotes MEKK2 activation and reveal the existence of a novel bi-directional regulatory relationship between MEKK2 and paxillin.

## Background

MEKK2 is a serine-threonine kinase within the MAP kinase kinase kinase (MAP3K) group of intracellular signaling regulatory enzymes. MEKK2 activity controls activation of the JNK and ERK5 MAP kinases [1,2], and as such is a component of a signaling node that regulates the expression and activity of multiple transcription factors, including activating protein 1 (AP1), NFkappa B, and MEF2C [3,4,5,6]. These factors, in turn, control the expression of numerous genes including cytokines and proteases [7,8,9,10]. Consistent with this functional profile, initial MEKK2 investigations linked MEKK2 to immune function and cytokine expression [11,12]. More recently, our group has revealed surprising functions for MEKK2 in control of tumor cell migration [13], xenograft tumor growth and metastasis [14]. Furthermore, Brown and colleagues have demonstrated that MEKK2-deficient mice are resistant to the development of hypertension-induced ventricular hypertrophy [15], suggesting that MEKK2 may be an important factor in cardiac disease. These recent reports indicate that the total spectrum of MEKK2 function and significance is likely to be much greater than was first imagined.

The scaffold protein paxillin functions as a key component of intracellular protein complexes called focal adhesions that link signaling and structural proteins to membrane-bound integrin receptors to transduce signals initiated by cellular attachment [16]. We previously reported that MEKK2 associates with paxillin to promote paxillin ubiquitylation and subsequent removal from focal adhesions [17]. Paxillin contains five leucine-rich “LD motifs” within its amino terminal half that mediate interaction with multiple signaling proteins [16,18,19], and we found that the LD1 motif is necessary for association with MEKK2 [17]. Although we established that MEKK2 regulates paxillin function, the impact of paxillin on MEKK2 function has not been determined.

Although our recent work has begun to elucidate MEKK2 function, and suggest that MEKK2 represents an important therapeutic target, little is known about the molecular mechanisms that control MEKK2 activity. In contrast to full-length MEKK2, the Phox and Bem1 (PB1) domain, located near the MEKK2 amino terminus, has been the subject of both functional and structural investigation. For example, Nakamura and colleagues demonstrated that the PB1 was essential for MEKK2 to bind and activate the substrate kinase MEK5, and that this interaction was necessary for MEKK2-dependent ERK5 activity [20]. However, PB1 domain-dependent functions other than binding MEK5 remain undefined. Herein, we report our evidence of a bi-directional regulatory relationship between paxillin and MEKK2, and the novel role of the PB1 domain in that interaction.

## Results

### Paxillin interaction induces MEKK2 auto-phosphorylation

**Figure 1:**
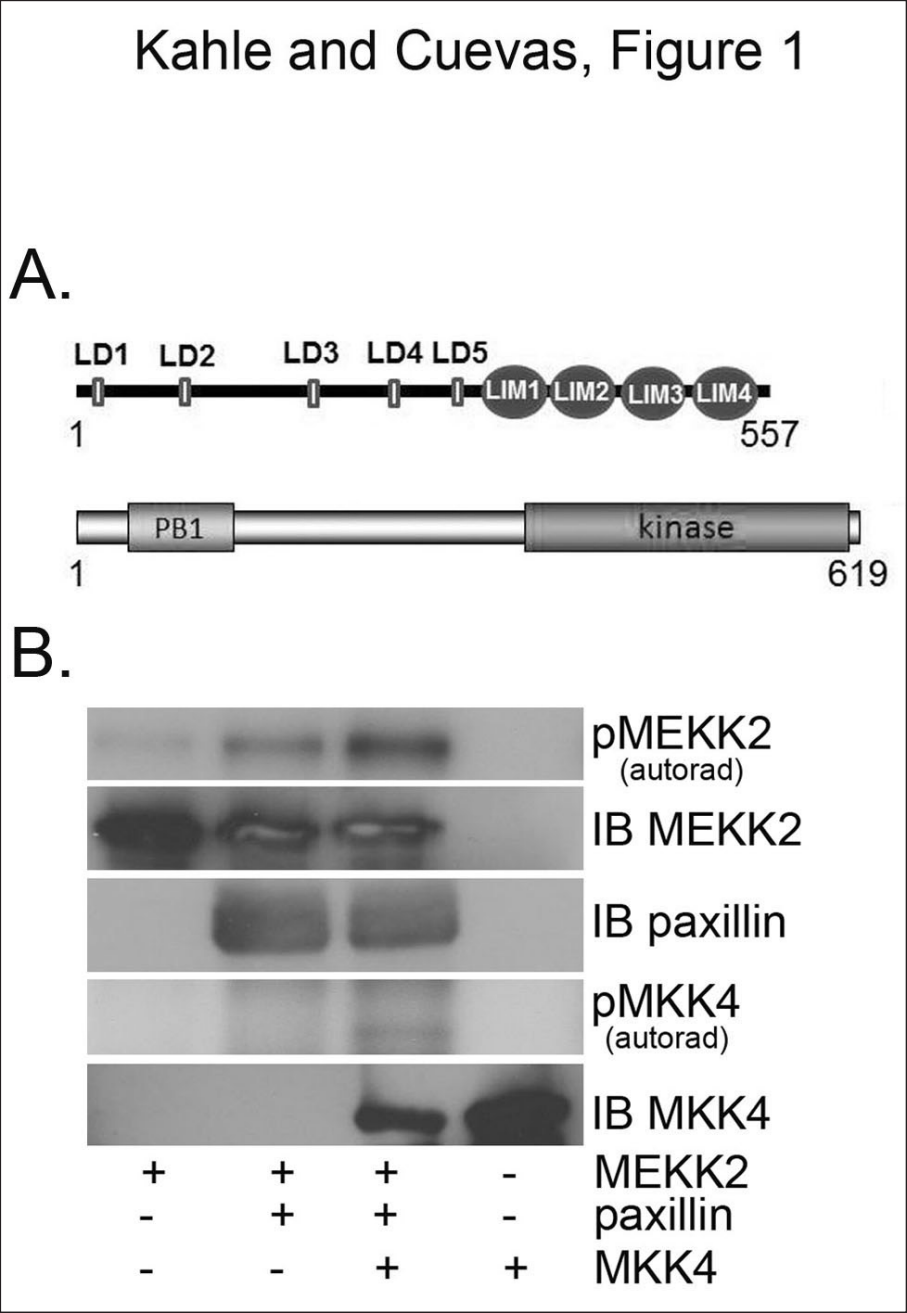
**Paxillin promotes MEKK2 kinase auto-phosphorylation.** A graphical representation of the domain structures of both MEKK2 and paxillin is shown in (A). (B) *In vitro* MEKK2 kinase assay analysis, with autoradiograph showing MEKK2 auto-phosphorylation (top panel), and MEKK2-dependent MKK4 phosphorylation (fourth panel). Anti-MEKK2 (second panel) and anti-paxillin (third panel) immunoblots were performed to demonstrate loading of reaction components. Results are representative of at least three independent experiments.

We previously reported that MEKK2 promotes paxillin ubiquitylation and that MEKK2 kinase activity is required for that function. Since phosphorylation has been shown to target proteins for ubiquitylation in some systems [21], we asked whether MEKK2 phosphorylates paxillin. To directly address this question, we performed an *in vitro* kinase assay with recombinant MEKK2 using recombinant paxillin as a substrate. While recombinant MEKK2 did not detectably phosphorylate paxillin in our experiments, we were surprised to discover that the addition of paxillin markedly enhanced MEKK2 phosphorylation (Figure 1B). As paxillin is a scaffold protein that does not display kinase activity, the MEKK2 phosphorylation observed must be due to auto-phosphorylation. This enhanced MEKK2 auto-phosphorylation was accompanied by only modest phosphorylation of known MEKK2 substrate MKK4, suggesting that paxillin induced in MEKK2 a conformation permitting auto-phosphorylation but not full activation; a state we called “permissive”. Conversely, the very low level of auto-phosphorylation displayed by recombinant MEKK2 without paxillin implies that MEKK2 can adopt an inactive (inhibited) conformation in the absence of activating stimuli, and that interaction with paxillin releases MEKK2 from this inhibited state. One consequence of MEKK2 adopting a paxillin-induced permissive conformation that may influence MEKK2 activation in cells may be that more and/or different phosphorylation sites are exposed to upstream regulator kinases than in the inhibited state, thus the permissive state facilitates full activation. Alternatively, docking motifs utilized by MEKK2 regulators may become more accessible in the permissive conformation than in the inhibited conformation. If this model of MEKK2 activity linked to paxillin-induced conformation is valid, our discovery represents an important advance in our understanding of the regulation of MEKK2 activity, and therefore we further investigated the role of paxillin in MEKK2 activation.

### Paxillin knockdown inhibits MEKK2 activity

**Figure 2:**
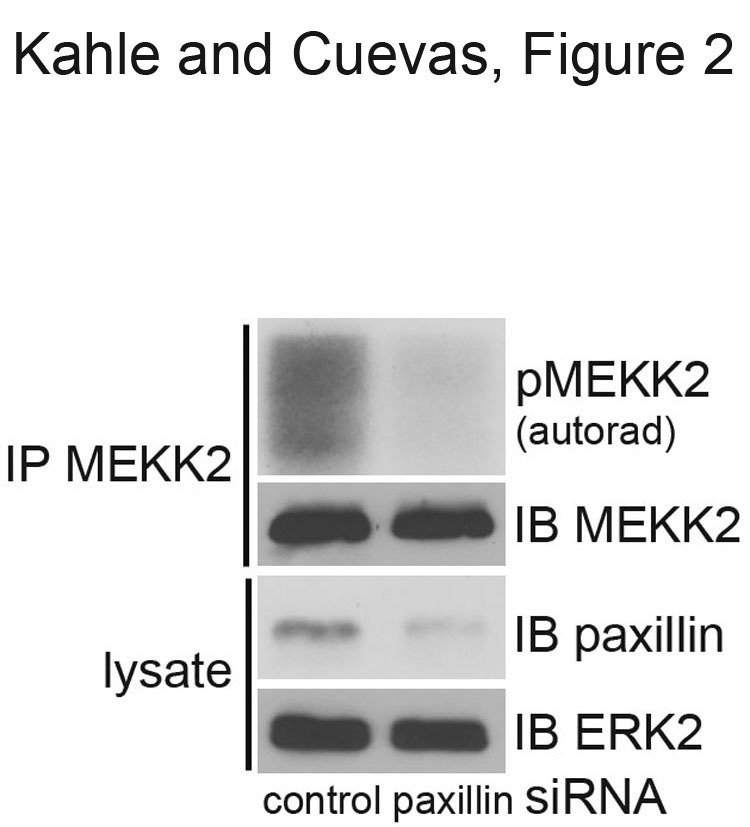
**Paxillin expression knockdown inhibits MEKK2 activity.** Displayed is an MEKK2 *in vitro* kinase assay with 32P ATP using MEKK2 auto-phosphorylation as an indication of kinase activity (top panel). MEKK2 was immunoprecipitated from 293T cells transfected with either paxillin siRNA or control siRNA. Anti-MEKK2 immunoblot shows the total amount of MEKK2 immunoprecipitated (second panel). Anti-paxillin immunoblot (third panel) shows siRNA-mediated expression knockdown, and anti-ERK2 blot demonstrates equal loading of lysate protein (bottom panel). Results are representative of at least three independent experiments.

To determine whether paxillin is required for MEKK2 activity in cells, we knocked down endogenous paxillin expression in HEK293T cells using a combination of four specific siRNA oligos. Using this approach we were able to consistently achieve a partial knockdown of paxillin expression by 48 hours post-transfection (Figure 2). Despite the incomplete knockdown, we observed that MEKK2 auto-phosphorylation was consistently reduced in the cells with lowered paxillin expression (Figure 2, top panel). These results indicate that paxillin knockdown is associated with a marked reduction in MEKK2 auto-phosphorylation, and strongly suggest that paxillin association with MEKK2 plays an important role in the regulation of MEKK2 activity.

### Paxillin interacts with the MEKK2 amino terminus

**Figure 3:**
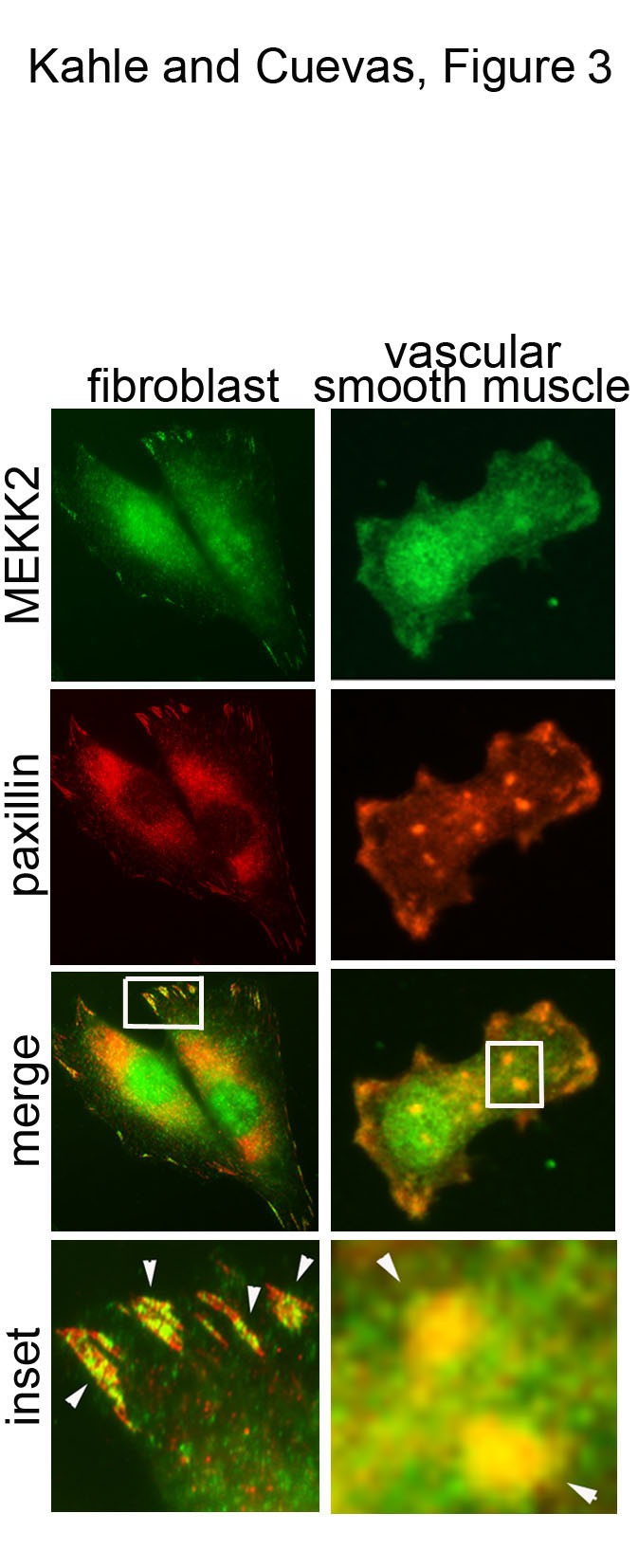
**Fibronectin-induced co-localization of MEKK2 and paxillin in diverse cell types.** Attachment to fibronectin induces MEKK2 to co-localize with paxillin in both murine fibroblasts (left column) and rat vascular smooth muscle cells (A7r5, right column). Cells were seeded on coverslips coated with fibronectin and allowed to attach for 6 hours, fixed and stained for immunofluorescence analysis with anti-MEKK2 (green) or anti-paxillin (red) antibodies. Arrowheads indicate areas of MEKK2/paxillin co-localization. Images are representative of at least three independent experiments.

We previously demonstrated that MEKK2 associates with paxillin in invasive breast cancer cells, and paxillin knockdown in HEK293T cells reduced MEKK2 activity, suggesting that paxillin binds MEKK2 in multiple cell types. To test this hypothesis, we performed immunofluorescence analysis to determine attachment-induced localization of endogenous MEKK2 in both murine fibroblasts and the vascular smooth muscle cell line A7r5. In each experiment, dispersed cells were allowed to attach to fibronectin-coated coverslips, fixed, permeabilized and both MEKK2 and paxillin were detected by specific antibodies. Similar to our observations in breast cancer cells, cellular attachment to fibronectin induces MEKK2 to co-localize with paxillin in areas of the cells that are consistent with focal adhesions (Figure 3). These data strongly suggest that MEKK2 association with paxillin is not limited to tumor cells, and occurs in multiple diverse cell types.

**Figure 4:**
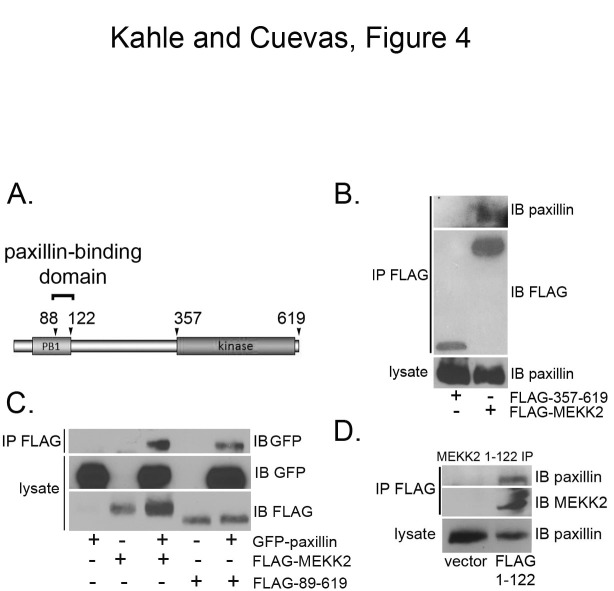
**Mapping the paxillin binding site in MEKK2.** (A) Diagram of MEKK2 protein with arrowheads indicating termini of truncation proteins used in these experiments. (B) Immunoblot analysis shows paxillin co-immunoprecipitating with FLAG-MEKK2 or FLAG-357-619 MEKK2. FLAG-tagged MEKK2 proteins were immunoprecipitated with anti-FLAG and co-immunoprecipitated paxillin was detected by anti-paxillin immunoblot (top panel). Endogenous paxillin present in each lysate was confirmed by anti-paxillin immunoblot (bottom panel), whereas the immunoprecipitated FLAG-tagged protein expression was detected by anti-FLAG blot (second panel). (C) FLAG-MEKK2 or FLAG-89-619 were co-transfected with GFP-paxillin, and the MEKK2 proteins were immunoprecipitated with anti-FLAG. Co-immunoprecipitated paxillin (top panel) was detected with anti-GFP blot. Expression of transfected proteins was confirmed with anti-FLAG and anti-GFP blots (second and bottom panels). (D) Cells were transfected with either empty vector or FLAG MEKK2 1-122, followed by immunoprecipitation of FLAG MEKK2 1-122 with anti-FLAG monoclonal antibodies. Co-immunoprecipitated paxillin from each lysate was detected by anti-paxillin immunoblot (top panel), as was total paxillin protein present in each lysate (bottom panel). Anti-FLAG immunoblot shows immunoprecipitated FLAG-1-122 (second panel). Results are representative of at least three independent experiments.

To ascertain how paxillin enhances MEKK2 activity, we examined the nature of the interaction between the two proteins. We created expression vectors encoding full-length and FLAG-tagged truncated MEKK2 proteins (Figure 4A) and then performed immunoprecipitation analysis to define the MEKK2 domain(s) required to interact with paxillin. We found that paxillin does not detectably associate with the kinase domain (Figure 4B), but does readily co-immunoprecipitate with a truncated MEKK2 that does not include the amino-terminal 88 amino acids of full-length MEKK2 (Figure 4C). Further examination revealed that paxillin binds an amino terminal fragment that includes the PB1 domain (amino acids 1-122, Figure 4D). These data show that neither the extreme MEKK2 amino terminus (amino acids 1-88) nor the kinase domain are required for MEKK2 to bind paxillin, and that the paxillin-binding region of MEKK2 is contained within the PB1 domain between amino acids 89-122.

### Paxillin LD1 peptide activates MEKK2 in vitro

**Figure 5:**
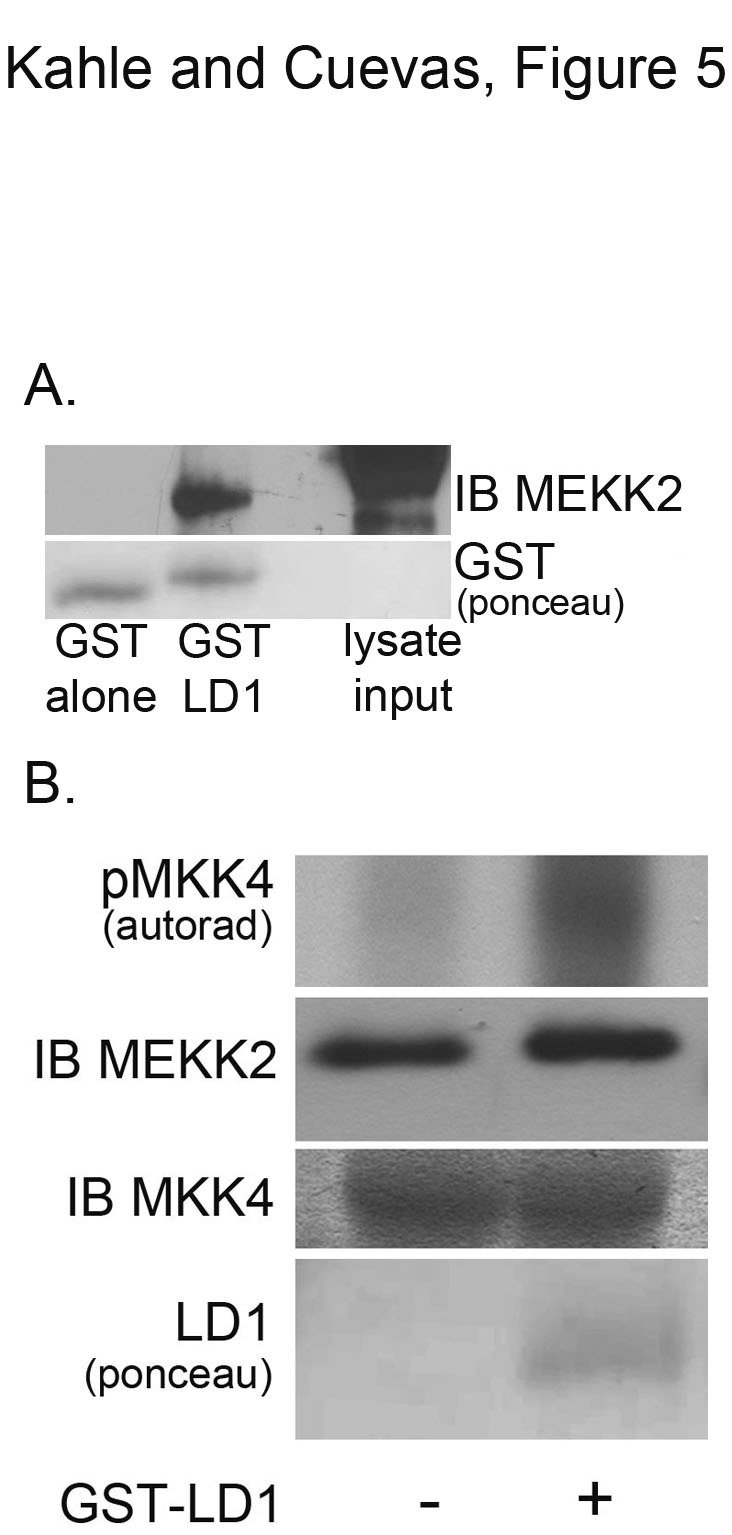
**Paxillin LD1 motif peptide induces MEKK2 activity *in vitro*.** LD1-GST pulldown of FLAG-MEKK2 from transfected 293T cell lysates is shown in (A) (upper panel) compared to an identical concentration of lysate pulled down with GST alone. Ponceau staining shows relative amounts of GST proteins used in the pulldown (lower panel). (B) MEKK2 *in vitro* kinase assay, with autoradiograph (top panel) showing rMKK4 phosphorylation +/- LD1-GST. FLAG-MEKK2 was immunoprecipitated from transfected cells with anti-FLAG antibodies and the activity assessed by *in vitro* kinase assays. Immunoblot analysis shows equal loading of MEKK2 (middle panel) and rMKK4 (bottom panel). Ponceau staining shows LD1-GST proteins included in the reaction (lower panel). Results are representative of at least three independent experiments.

To determine whether the LD1 motif alone is sufficient to mediate interaction with MEKK2, we utilized purified recombinant GST-LD1 fusion proteins to pull down MEKK2 from transfected cell lysates. We observed that LD1 peptide efficiently captured MEKK2 from lysates (Figure 5A), indicating that the paxillin LD1 is indeed necessary and sufficient to mediate the interaction between paxillin and MEKK2. To determine whether LD1 binding was sufficient to activate MEKK2 expressed in mammalian cells, we performed *in vitro* kinase assays with MEKK2 protein immunoprecipitated from transfected HEK293Tcells and included recombinant LD1 in the assay reaction mixture along with the MKK4 substrate. We found that MKK4 phosphorylation was increased in the presence of LD1 peptide, indicating that MEKK2 kinase activity was enhanced by LD1 peptide (Figure 5B). Our results strongly suggest that LD1 association with MEKK2 was sufficient to enhance MEKK2 activity.

## Discussion

In this study, we present evidence that physical association with the scaffold protein paxillin promotes MEKK2 activation. Combined with our previous report that MEKK2 regulates paxillin ubiquitylation, our data strongly suggest that a bi-directional regulatory relationship exists between MEKK2 and paxillin, with each protein influencing the function of the other. While paxillin is well established as an important signaling scaffold, to our knowledge this is the first report to show that paxillin binding alone alters activity of a MAP3K.

MEKK2 activity has been linked to physiological functions ranging from immune function to regulation of bone mass, and therefore plays important roles in homeostasis of multiple organ systems. Furthermore, animal models of disease suggest that ablation of MEKK2 expression blocks hypertension-induced cardiac hypertrophy and breast xenograft tumor metastasis, leading to considerable interest in MEKK2 as a therapeutic target. At present, selective MEKK2 inhibitors have not been reported. A major challenge in kinase drug discovery and development is to develop small molecule inhibitors that specifically bind the kinase domain of one kinase only, when the targeted kinase domain contains conserved features found in the kinase domains of many related kinases. This obstacle underscores the need to define regulatory interactions required for kinase activity that can be targeted with greater selectivity that the catalytic cleft of the kinase domain.

Our kinase assay analysis revealed that the modest activity of recombinant MEKK2 activity is markedly enhanced by the presence of paxillin. This finding suggests that recombinant MEKK2 activation includes the following characteristics. First, as paxillin has neither a known catalytic domain nor enzymatic activity, paxillin cannot modify MEKK2 protein by attaching phosphor, acetyl, methyl or other molecules that has been shown to alter kinase activity. As our experimental conditions included recombinant paxillin and MEKK2 only, without other potential MEKK2 regulators in the reaction, it follows that paxillin changes MEKK2 activity by physical association alone. This observation implies that non-stimulated MEKK2 adopts a conformation characterized by low kinase activity in the absence of paxillin. We would hypothesize that association with paxillin relieves this inhibited conformation and induces MEKK2 to adopt a permissive conformation that is conducive to full activation when other essential activators are present. We have shown that paxillin association with MEKK2 requires the amino acids within the LD1 motif (LDALLADL) that have been reported to form a helix with leucine residues aligning to form a hydrophobic surface [22]. By what mechanism could a short, leucine-rich peptide relieve MEKK2 auto-inhibition? Our hypothesis is that the PB1 domain is required for MEKK2 to adopt the inhibited conformation, and that paxillin binding to the PB1 domain via LD1 induces release of MEKK2 from the inhibited state. By discovery of a key regulatory sequence that is outside the kinase domain, our work reveals an exciting opportunity to rationally design chemicals that inhibit MEKK2 by blocking the interaction with paxillin. Future studies will focus on identifying the crucial motif within the PB1 domain that is required to bind LD1 in order to clarify the mechanism by which paxillin promotes MEKK2 activity.

## Conclusions

In this manuscript, we reveal that paxillin interaction promotes an active state in the kinase MEKK2. The paxillin LD1 motif is sufficient for both interaction with MEKK2 and induction of MEKK2 auto-phosphorylation. Therefore, we propose a model of paxillin-dependent MEKK2 activity in which paxillin interaction with MEKK2 via the LD1 motif binding to the MEKK2 PB1 domain releases MEKK2 from its auto-inhibitory state and thus primes MEKK2 for further activation.

## Methods

### Antibodies and reagents

Anti-MEKK2 and ERK2 antibodies were purchased from Santa Cruz Biotechnology. Anti-FLAG antibodies were purchased from Sigma. Protein G agarose beads were purchased from Roche Applied Science. Anti-paxillin antibodies were purchased from BD Biosciences. Anti-phospho-MKK4 antibodies were purchased from Cell Signaling Technology. Recombinant MEKK2 was purchased from SignalChem, and recombinant paxillin was purchased from RayBiotech, Inc.

### Plasmid vectors

The cDNA vectors encoding wild-type FLAG-tagged and HA-tagged MEKK2 were previously described [13,17]. The FLAG-tagged expression vectors encoding the MEKK2 truncation mutants were produced by PCR and cloned into FLAG/pCDNA3. The bacterial expression vectors encoding GST-LD1 was a generous gift of Dr. Jim C. Norman (Beatson Institute for Cancer Research, Glasgow, UK).

### Immunofluorescence microscopy

MEKK2 localization studies by immunofluorescence microscopy analysis were performed as previously described [7].

### Paxillin knockdown

Endogenous paxillin expression was knocked down in HEK-293T cells by transfecting specific siRNA. 0.2 nmol of pooled ON-TARGET plus Human PXN siRNAs (Dharmacon/GE SMARTpool, J-005163, oligos 5-8) were mixed with Oligofectamine (Invitrogen) in low-serum media (OptiMEM, Invitrogen) before being added dropwise to cells in antibiotics-free media. Cells were lysed 48 hours post-transfection and analyzed as indicated.

### Cell culture and transfection

HEK-293T cells were purchased from A.T.C.C. Cells were cultured in DMEM (Dulbecco’s modified Eagle’s medium) (Invitrogen) containing 10% (v/v) fetal bovine serum (Atlanta Biologicals) at 37°C and maintained in 5% CO2 in a humidified atmosphere. A7r5 rat cells were a generous gift of Dr. Kenneth Byron (Loyola University Chicago), and the mouse embryo fibroblasts used in this study were previously described [23]. All transfections were conducted using linear polyethylenimine (PEI, MW 25000, Polysciences Inc., Warrington, PA). Briefly, PEI was dissolved in water and mixed with DNA vectors at a 3:1 ratio (μg/μg) in OptiMEM (Life Technologies) and allowed to form complexes for 20 minutes at room temperature prior to adding the complexed DNA dropwise to cells in culture. Cells were harvested 48 hours post-transfection.

### MEKK2 kinase assay

*In vitro* kinase activity assays of recombinant or transfected MEKK2 were performed as previously described for endogenous MEKK2 [13]. Where indicated, 1 μg of recombinant MEKK2 was assayed +/- 1 μg of recombinant paxillin. Incorporation of phosphorous from 32P ATP (PerkinElmer) was used as a indicator of MEKK2 auto-phosphorylation, and MKK4 phosphorylation was detected by immunoblots using anti-phospho-MKK4 antibodies.

## Competing interests

The authors declare that they have no competing interests.
